# Still too little qualitative research to shed light on results from reviews of effectiveness trials: A case study of a Cochrane review on the use of lay health workers

**DOI:** 10.1186/1748-5908-6-53

**Published:** 2011-05-27

**Authors:** Claire Glenton, Simon Lewin, Inger B Scheel

**Affiliations:** 1 Department of Global Health and Welfare, SINTEF Society and Technology, Oslo, Norway; 2 Norwegian Knowledge Centre for the Health Services, Oslo, Norway; and Medical Research Council of South Africa

## Abstract

**Background:**

Qualitative research is used increasingly alongside trials of complex interventions to explore processes, contextual factors, or intervention characteristics that may have influenced trial outcomes. Qualitative research conducted alongside trials can also be used to shed light on the results of systematic reviews of effectiveness by looking for factors that can help explain heterogeneous results across trials. In a Cochrane review on the effects of using lay health workers on maternal and child health and infectious disease control, we identified 82 trials. These trials showed promising benefits but results were heterogeneous.

**Objective:**

To use qualitative studies conducted alongside these trials to explore factors and processes that might have influenced intervention outcomes.

**Methods:**

We attempted to identify qualitative research carried out alongside the trials by contacting trial authors, checking papers for references to qualitative research, searching Pubmed for related studies, and carrying out citation searches. For those qualitative studies that we included, we extracted information regarding study objective, data collection and analysis methods, and key themes and categories.

**Results:**

For 52 (63%) of the trials, we found no qualitative research that had been conducted alongside the trials. For 16 (20%) trials, some form of qualitative data collection had been done but was unavailable or had been done before the trial. For 14 (17%) trials, qualitative research had been done during or shortly after the trial, although descriptions of qualitative methods and results were often sparse. Most of these 14 studies aimed to elicit trial participants' perspectives and experiences of the intervention. A common theme was participants' appreciation of the lay health workers' shared circumstances, for instance with regard to social background or experience of the health condition. In six studies, researchers explored the experiences of the lay health workers themselves. Issues included the importance of regular supervision and health professionals' support or lack of support.

**Conclusions:**

Qualitative studies carried out alongside trials of complex interventions could offer opportunities to authors of systematic reviews of effectiveness wishing to understand the heterogeneity of trial results. For interventions of lay health worker programmes at least, too few such studies exist at present for these opportunities to be realised.

## Background

Interventions that aim to improve the organisation and delivery of healthcare often involve complex socio-behavioural processes, and are frequently 'made up of various interconnecting parts' [1] that act both 'independently and inter-dependently' [2,3], and that may be highly context-dependent [4]. There is growing acknowledgement of the contribution that qualitative research can make to both the development and evaluation of these complex interventions, and randomised trials of such interventions are increasingly including qualitative components [5,6]. Qualitative research can be used prior to a trial of a health system intervention to increase the quality and relevance of the intervention and to help select relevant outcomes, but can also be used during or after a trial to explore processes, contextual factors or intervention characteristics that may have influenced the trial results. In principle, qualitative research can also be used to shed light on the findings of systematic reviews of the effectiveness of health system interventions by looking for processes and other factors that could help explain homogeneous or heterogeneous results across trials or that could suggest new sub-group analyses for reviews.

In a recent Cochrane review on the effects of using lay health workers for maternal and child health and infectious disease control [7], we identified 82 randomised trials. These trials showed promising benefits in a number of areas, including in the use of lay health worker programmes to increase breastfeeding and childhood immunization. However, the results within these subgroups were heterogeneous. We wished to explore this heterogeneity by reviewing whether qualitative research conducted alongside these trials could increase our understanding of the processes that took place in these trials as well as contextual factors potentially influencing the outcomes of the intervention.

## Objective

Our objective was to use qualitative studies conducted alongside randomised trials of lay health worker programmes included in a Cochrane review to explore the factors and processes that might have influenced the outcomes of these programmes.

## Methods

We attempted to identify published and unpublished qualitative research carried out alongside the trials included in the Cochrane review. We defined a qualitative study as any study that used qualitative methods for data collection and analysis. We contacted the authors of the 82 trials, asking if any such research had taken place. For the 26 trials where no response was forthcoming, one researcher (CG) checked the main text and the reference list of each trial for descriptions of, or references to, related qualitative research; located each trial in Pubmed and searched for related studies and for other studies published by the same authors; and located each trial in the Science and Social Science Citation Index and checked the list of studies that had cited this paper. The same researcher then assessed full versions of potential papers to determine whether they were related to the trial and whether they had used qualitative research methods. For those studies that were included, we extracted information regarding the objective of the qualitative study, the methods of data collection and analysis used, and the key themes and categories identified.

## Results

**Figure 1:**
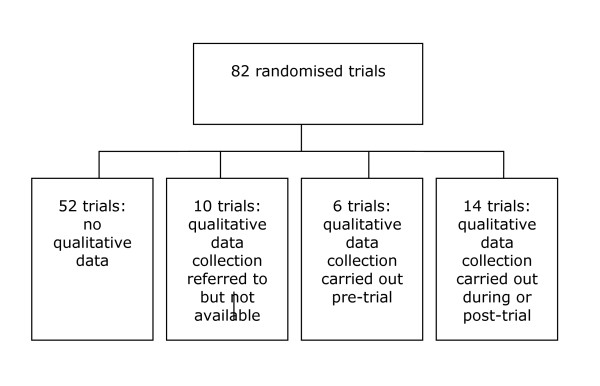
**Flow chart**.

Fifty-two (63%) of the 82 trials had no qualitative research linked to them. For ten (12%) of the trials, some form of qualitative data collection was referred to briefly in the paper or in emails from authors, but was unavailable. At least half of this research appeared to have been done before the trial in order to develop the intervention. For a further six (7%) of the trials, qualitative research had been carried out before the trial and was available as either published or unpublished reports. The aim of these studies was to help develop the intervention by exploring the study population's health knowledge and behaviour, factors that influenced this behaviour, experiences of illness and healthcare, or healthcare needs. While these studies may have been important to the development of the trialed intervention, they did not allow us to explore directly the processes or other factors that may have influenced the outcomes of the trials and were therefore not explored further (See also Figure 1).

**Figure 2:**
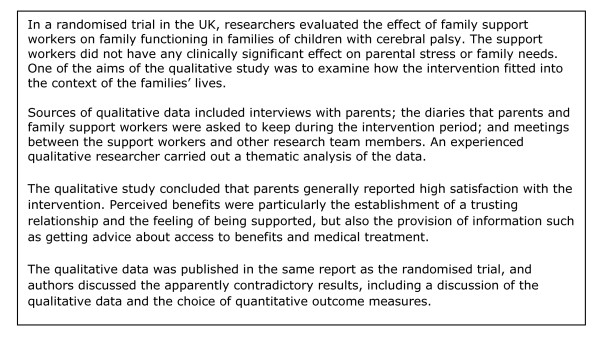
**Example of a qualitative study carried out alongside a randomised trial: lay health workers for families of children with cerebral palsy (Adapted from Weindling 2007 **[19]).

**Figure 3:**
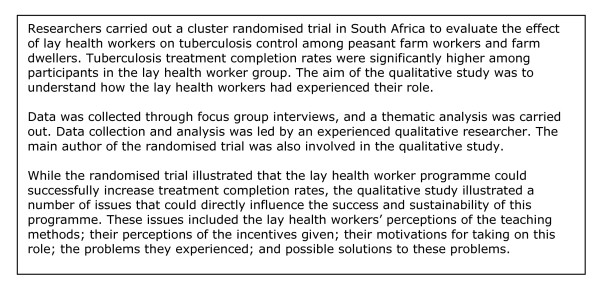
**Example of a qualitative study carried out alongside a randomised trial: lay health workers for people with tuberculosis (Adapted from Clarke et al 2005 **[17]**and Daniels et al 2005 **[29]).

For 14 (17%) of the 82 trials [8-21], qualitative data collection had been carried out during or shortly after the trial, or, in one case, after the pilot study for the trial (See Figure 2 and Figure 3 for examples). For four trials [10,12,16,19], these data were presented in the same paper as the trial, while for one trial, these data were presented both in the same paper and in a separate paper [10,22]. For the remaining ten trials [8,9,11,13-15,17,18,20,21], qualitative data were presented separately, and in most cases published [23-32] and also cross-referenced with the trial publications. Descriptions of qualitative methods and results were often sparse, particularly for six of the studies [12,16,23-25,31] where authors offered little or no information about data collection methods and/or data analysis. In at least four of these six cases, the qualitative data were not the only focus of the paper.

In these 14 trials, lay health worker programmes had been used to support women with poor pregnancy outcomes or families with sick children, to promote breastfeeding, to improve tuberculosis-related outcomes, to reduce child mortality and morbidity, and to prevent child injuries in the home. The trials were conducted in the USA (five studies), UK (three studies), South Africa (two studies), Bangladesh (two studies), Ghana and Nepal, and generally made use of lay health workers who were local to the setting and who had been selected on the basis of their similarity to the trial participants, for instance with regard to illness experiences.

The qualitative studies either looked at the perspectives of trial participants (eight studies); lay health workers (one study); or both (five studies). A common theme among trial participants was their appreciation of the similarities between them and the lay health workers, for example with regard to social background or because of first-hand experience of the health behaviour in question (breastfeeding) or the health condition (children with a particular illness). These similarities represented to participants an opportunity for emotional support as lay health workers similar to them were seen as being more accepting of participants' thoughts and actions [22]. These similarities were also seen as a source of practical support as these lay health workers would 'know all the pitfalls' [19]. One of the studies describes how participants who did not find the lay health worker programme helpful often pointed to factors associated with a lack of 'perceived sameness,' for example because of differences between lay health workers and study participants regarding illness experiences or preferences and values [22]. Participants across studies also described a number of other characteristics they regarded as important for a lay health worker, including patience and persistence, compassion and tolerance, accessibility, knowledge and common sense.

The shared experiences of the lay health workers and the trial participants were also valued by lay health workers in these studies. In addition, the lay health workers highlighted other issues including the importance of regular supervision and their experiences of support, or lack of support, from health professionals and the community in which they were based. One study of South African farm dwellers' experiences of becoming lay health workers illustrates how the transition from peer to lay health worker, and the new relationships this created with project staff, farm owners, and health professionals, led to mistrust and criticism from their family and the community [29].

## Discussion

Randomised trials are considered the most rigorous design for evaluating whether an intervention is effective. However, trials generally yield limited insights into intervention mechanisms [33], and other approaches are therefore needed to understand how the intervention was delivered and why it achieved the outcomes that it did, and indeed to assess whether the outcomes measured were the most appropriate ones [33]. These types of questions are particularly pertinent for interventions intending to change the organisation or delivery of healthcare, where a broader understanding of process is necessary if we are to understand the intervention's success or failure. For lay health worker programmes, the wider inclusion of qualitative research alongside the trials would have allowed us to explore a number of factors that may have influenced programme outcomes. These include factors associated with the programme itself, such as how the lay health workers were selected and trained and their relationship with communities and with professional health workers; but also the broader context of the programme, such as political, social or cultural conditions.

Qualitative studies of lay health worker programmes can also be carried out independently of trials of interventions. Such studies have described a range of issues that may influence programme sustainability and success, including factors that affect lay health worker motivation and retention (for instance [34-37]). But our goal was to expand our understanding of the trial interventions included in the Cochrane review, to see if certain patterns would emerge that could help us to understand the heterogeneity of the review results. However, only 14 of the trials had carried out some form of qualitative data collection during or after the intervention. These data suggest that perceived similarities between trial participants and lay health workers are seen as important by these groups. The identification of factors such as these may offer a basis for subgroup analyses in the Cochrane review, and may help explain heterogeneity in trial results. In general, however, the data we identified was sparse, and methods and results were often poorly described, making our study aim difficult to achieve.

This work reflects findings from an earlier study, where we examined the use of qualitative approaches alongside randomized trials of complex health service interventions [38]. In a sample of 100 trials, only 30 had associated qualitative work, around one-half of which had been carried out before the trial. Factors that may influence whether qualitative studies are done alongside trials include the attitudes of funding bodies and the attitudes and skills of the research community [39]. When mixed methods are used, lack of time or experience as well as journal formats may prevent findings from qualitative studies and trials or reviews of effectiveness from being integrated or presented together [39]. The revision of formats for trial and review reporting is one way forward, and electronic publication now creates opportunities for publication of supplementary materials providing further detail regarding qualitative and other studies conducted alongside trials. Journals encouraging mixed methods will also, however, need to ensure that these papers receive appropriate peer reviewing. In addition, qualitative studies and trials that are reported separately need to be more clearly linked to one another to facilitate retrieval. All trials now require a universal trial reference number, and qualitative studies carried out alongside trials should utilize this number to facilitate linkage. Electronic publication databases could also utilise these reference number to show linked groups of studies when any one of the studies are retrieved.

## Conclusion

Qualitative studies carried out alongside trials of complex health system interventions could offer insights into intervention mechanisms, and give authors of systematic reviews of effectiveness an opportunity to explore the reasons for heterogeneity among trial results [38,40]. For interventions involving lay health workers at least, too few such studies exist at present for these opportunities to be realised. Those conducting trials of lay health worker programmes should incorporate in-depth process evaluation, including qualitative analysis to explore the reasons for the outcomes of these complex interventions. Methodological and practical guidance may be needed for trial teams who plan to use qualitative approaches for this purpose.

## Competing interests

The authors declare that they have no competing interests.

## Authors' contributions

CG, SL and IBS conceived of and designed the study. CG searched for and assessed the studies and drafted the manuscript. SL assessed papers where there was doubt regarding inclusion or allocation. CG drafted the paper and the other authors then contributed to this. All authors read and approved the final manuscript.
